# A1 adenosine receptor attenuates intracerebral hemorrhage-induced secondary brain injury in rats by activating the P38-MAPKAP2-Hsp27 pathway

**DOI:** 10.1186/s13041-016-0247-x

**Published:** 2016-06-14

**Authors:** Weiwei Zhai, Dongdong Chen, Haitao Shen, Zhouqing Chen, Haiying Li, Zhengquan Yu, Gang Chen

**Affiliations:** Department of Neurosurgery & Brain and Nerve Research Laboratory, The First Affiliated Hospital of Soochow University, 188 Shizi Street, Suzhou, 215006 China

**Keywords:** Adenosine A1 receptor, Intracerebral hemorrhage, P38, MAPKAP2, Hsp27

## Abstract

**Background:**

This study was designed to determine the role of the A1 adenosine receptors in intracerebral hemorrhage (ICH)-induced secondary brain injury and the underlying mechanisms.

**Methods:**

A collagenase-induced ICH model was established in Sprague–Dawley rats, and cultured primary rat cortical neurons were exposed to oxyhemoglobin at a concentration of 10 μM to mimic ICH in vitro. The A1 adenosine receptor agonist N(6)-cyclohexyladenosine and antagonist 8-phenyl-1,3-dipropylxanthine were used to study the role of A1 adenosine receptor in ICH-induced secondary brain injury, and antagonists of P38 and Hsp27 were used to study the underlying mechanisms of A1 adenosine receptor actions.

**Results:**

The protein level of A1 adenosine receptor was significantly increased by ICH, while there was no significant change in protein levels of the other 3 adenosine receptors. In addition, the A1 adenosine receptor expression could be increased by N(6)-cyclohexyladenosine and decreased by 8-phenyl-1,3-dipropylxanthine under ICH conditions. Activation of the A1 adenosine receptor attenuated neuronal apoptosis in the subcortex, which was associated with increased phosphorylation of P38, MAPK, MAPKAP2, and Hsp27. Inhibition of the A1 adenosine receptor resulted in opposite effects. Finally, the neuroprotective effect of the A1 adenosine receptor agonist N(6)-cyclohexyladenosine was inhibited by antagonists of P38 and Hsp27.

**Conclusions:**

This study demonstrates that activation of the A1 adenosine receptor by N(6)-cyclohexyladenosine could prevent ICH-induced secondary brain injury via the P38-MAPKAP2-Hsp27 pathway.

## Background

Intracerebral hemorrhage (ICH) is a frequent cause of mortality (estimated mortality rate ~ 50 %) and morbidity (accounts for ~ 10–15 % of all strokes) during the perioperative period and may cause patient paralysis or even death [1, 2]. Progression of the pathophysiology of ICH is still not very well understood [3, 4]. However, there is a general consensus that hemorrhage in the brain [5] leads to tissue disruption and displacement [6]. A series of secondary pathophysiological processes, which are referred to as secondary brain injury (SBI) [7], includes ischemia of brain tissue surrounding the hematoma, development of brain edema [8, 9], activation of apoptotic programs [10, 11], and toxic effects of the hematoma [12–14]. Finding treatments that relieve the pathophysiology of ICH has challenged neurosurgeons for many years.

As a potent biological mediator, adenosine has been reported to be extensively released during hemorrhage and eliminated quickly from plasma by the uptake mechanism and adenosine deaminase [15]. And both the uptake and the deamination are effective in removing extracellular adenosine and regulate the activation balance of adenosine receptors [16]. All the four adenosine receptor subtypes, including A1, A2a, A2b and A3, can be stimulated by extracellular adenosine when its concentration reaches the micromolar range [17]. It has been reported that adenosine deaminase activity in the poor-grade subarachnoid hemorrhage (SAH) patients was higher than that in the good-grade SAH patients [18]. In recent years, evidence has shown that the adenosine receptors, particularly A2a adenosine receptor (A2aAR), are of critical importance in hemorrhagic stroke [19]. A2aAR has been shown to be involved in the beneficial effect of 17beta-estradiol in attenuating SAH-induced apoptosis and vasospasm [20]. And A2aAR agonism is effective in preventing SAH-induced vasospasm. However, global inactivation of A2aAR could confer protection against the early ischemic vascular injury after SAH, suggesting that early inhibition of A2aAR after SAH might reduce cerebral injury. As shown above, the previous studies are mainly focused on the role of A2aAR in SAH-induced brain injury, which is controversial and may be selectively manipulated by targeting different cellular elements [19]. Besides, the positive roles of A1AR in SAH-induced vasospasm [21] and A2aAR in ICH-induced proinflammatory events and cell death have been reported. However, the effects of the four adenosine receptor subtypes on ICH-induced SBI still remain elusive and must be clarified.

Mitogen-activated protein kinase (MAPK) family, including extracellular signal-regulated kinase (ERK), p38, and Jun N-terminal Kinase (JNK), widely mediates inflammation, cell proliferation, and apoptosis. As the activation of MAPK pathways by A1 [22, 23], A2a [22, 24, 25] and A2b [22, 26] and A3 [22] adenosine receptors has been demonstrated, the involvement of this intracellular phosphorylative cascade in adenosine receptor regulation has attracted more and more attention. A1AR mediated p38 MAPK activation plays a crucial role in the presynaptic inhibitory effect of adenosine on CA3–CA1 synaptic transmission [27]. A1AR-mediated p38 MAPK and JNK activation is a crucial step in regulating AMPAR trafficking during prolonged hypoxia [28]. Human adenosine A1, A2A, A2B, and A3 receptors expressed in Chinese hamster ovary cells all mediate the phosphorylation of ERK [23]. Elucidating the signaling pathway involved in the action of adenosine receptors will facilitate the understanding of death or survival mechanisms in ICH and the development of targeting drugs.

In addition, it has been pointed out that differences in the efficacy of adenosine receptors may be caused by differences in expression. Overexpressions of adenosine receptors can lead to signal transductions that do not occur in cells with the receptors expressed at a normal level [29]. In the present study, we tested the time course of the expression of the four adenosine receptor subtypes and evaluated the effects of ICH on these parameters. We hypothesized that pharmacological agonists selective for adenosine receptors would provide neuroprotection following ICH. Our results demonstrated that, among the four adenosine receptor subtypes, only the protein level of A1AR was significantly increased in rat brain after ICH and oxyHb-treated neurons, while the other three receptors have no significant changes. 48 h after ICH, activation of A1AR by R-PIA could prevent ICH-induced SBI via the p38-MAPKAP2-Hsp27 pathway.

## Results

### Time course

**Fig. 1:**
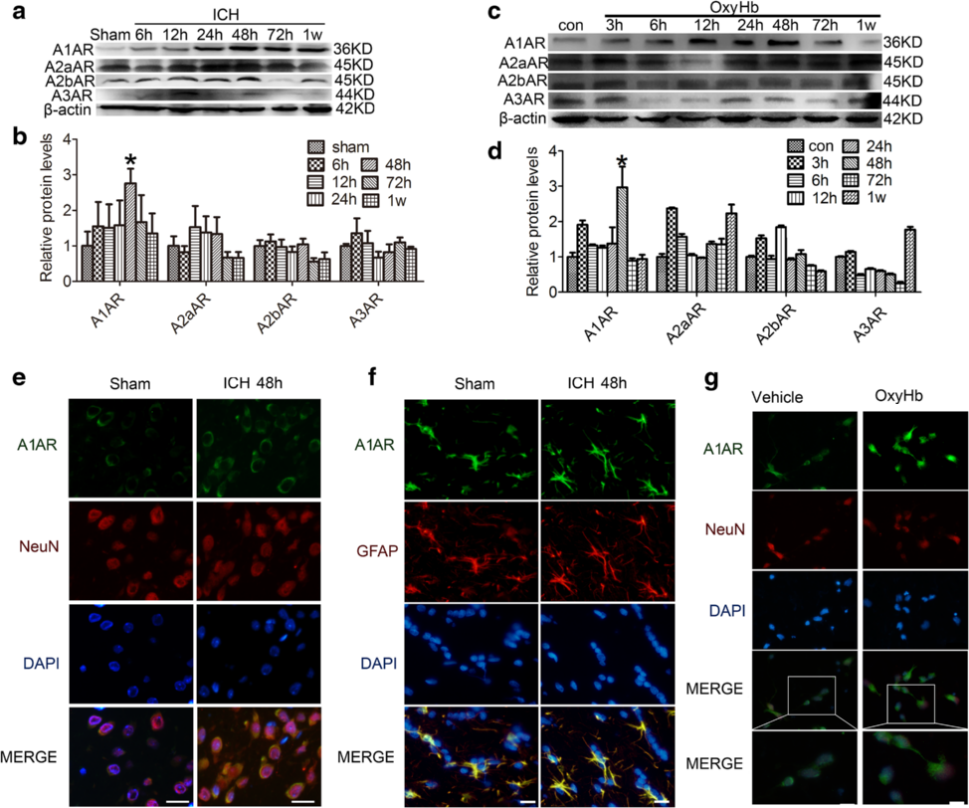
Changes in adenosine receptor expression in vivo and in vitro. **a** Western blot analysis of adenosine receptor expression at different time points following ICH in vivo. A1AR levels peaked at 48 h. **b** Quantification of the results in panel A. Bars represent relative protein levels. The mean values of the protein levels in the sham group were normalized to 1.0. *p < 0.05 for the 48 h ICH group versus the sham group. **c** Western blots of adenosine receptor expression in vitro. A1AR levels peaked at 48 h. **d** Quantification of the results in panel C. Bars represents relative protein levels. The mean values of the protein levels in the control group were normalized to 1.0. *p < 0.05 for the 48 h OxyHb group versus the control group. **e** and **f** Immunofluorescence in vivo*.* Double immunofluorescence analysis was performed with A1AR antibodies (green) and neuronal or astrocyte marker (NeuN/GFAP, red), and nuclei were fluorescently labeled with DAPI (blue). Scale bar = 32 μm. G: Immunofluorescence in vitro. Double immunofluorescence analysis was performed with A1AR antibodies (green) and neuronal marker (NeuN, red), and nuclei were fluorescently labeled with DAPI (blue). Scale bar = 20 μm

We first performed a time course experiment (Fig. 1). After establishment of the ICH model, rats were killed at the following time points after induction of ICH: 0 h, 6 h, 12 h, 24 h, 48 h, 72 h, and 1 week. In western blot analysis, compared with the other adenosine receptors, A1AR levels varied the most (Fig. 1a and b). In contrast to the group subjected to ICH, the sham group expressed a low level of A1AR. After induction of ICH, the level of A1AR increased with time, peaking at 48 h, and then decreased. Consistant with the in vivo data, western blot assay showed that the protein level of A1AR in cultured primary neurons was significantly increased after oxyHb-incubated for 48 h (Fig. 1c and d). Double immunofluorescence assay further verified the ICH-induced increase in the protein level of A1AR in neurons (Fig. 1e). In addition, Jeong et al. reported that astrogliosis participated in neuronal recovery in ATP-injected brain [30]. To pursue the potential role of astrogliosis in A1AR action in this ICH model, we also tested the protein level of A1AR in astrogliosis by double immunofluorescence. And the results showed that there was no significant change in the protein level of A1AR in astrogliosis between sham group and ICH group (Fig. 1f). Finally, double immunofluorescence also showed that oxyHb treatment could increase the protein level of A1AR in cultured neurons (Fig. 1g). So, we focused on A1AR in neurons in the following study.

### A1AR activation suppressed caspase-3 activation and albumin extravasation

**Fig. 2:**
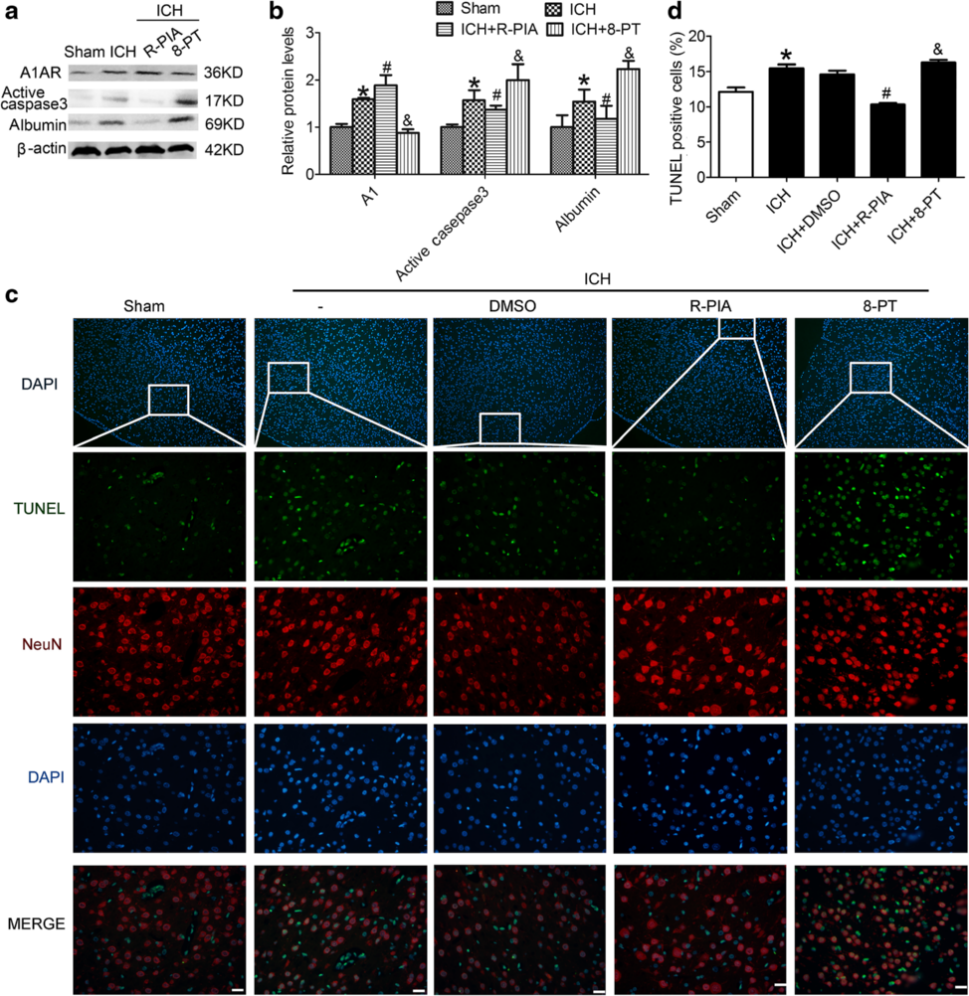
Effects of A1AR on ICH-induced SBI. **a** Western blot analysis showing expression of A1AR, active caspase-3, and albumin in the sham, ICH, ICH + R-PIA, and ICH + 8-PT groups at 48 h after ICH onsets. **b** Quantification of the results in panel A. The mean values of the protein levels in the sham group were normalized to 1.0. *p < 0.05 for the ICH group versus the sham group, ^#^p < 0.05 for the ICH + R-PIA group versus the ICH group, & p < 0.05 for the ICH + 8-PT group versus the ICH group. **c** TUNEL staining showing effects of A1AR on SBI at 48 h after ICH onsets. Representative images from sham, ICH, ICH + DMSO, ICH + R-PIA, and ICH + 8-PT groups. Each group was subjected to ICH except for the sham group. Scale bar = 50 μm. **d** The percentage of TUNEL-positive neurons. *p < 0.05 for the ICH group versus the sham group, ^#^p < 0.05 for the ICH + R-PIA group versus the ICH group, & p < 0.05 for the ICH + 8-PT group versus the ICH group

We conducted further studies using the A1AR agonist, N(6)-cyclohexyladenosine (R-PIA), and the A1AR antagonist, 8-phenyl-1,3-dipropylxanthine (8-PT). Both agonist and antagonist were administered 30 min before induction of ICH. Rats were randomly divided into 4 groups: sham group, ICH group, ICH + R-PIA group, and ICH + 8-PT group. We performed western blot analysis at 48 h after ICH onsets and detected changes in the protein levels of active caspase-3 and albumin (Fig. 2a and b). Protein levels of active caspase-3 and albumin showed significant increases in the ICH group compared with the sham group. Treatment with the agonist R-PIA suppressed the ICH-induced increase in levels of caspase-3 and albumin (p < 0.05). In contrast, treatment with the A1AR antagonist, 8-PT, enhanced ICH-induced upregulation of caspase-3 and albumin protein levels (p < 0.05).

### A1AR decreased neuronal death and degeneration and relieved brain edema

We evaluated neuronal death and degeneration using terminal deoxynucleotidyl transferase-mediated dUTP nick end labeling (TUNEL) and Fluoro-Jade B (FJB), respectively. Rats subjected to ICH or ICH + DMSO demonstrated histological evidence of neuronal death compared with the sham group (Fig. 2c and d), while there was no obvious differences observed between the ICH and ICH + DMSO groups. The group pretreated with R-PIA before ICH injury demonstrated a significant decrease in cell death ratio in the rat brain sample. In contrast, pretreatment with the A1AR receptor antagonist 8-PT before ICH injury led to an increase in the number of TUNEL-positive cells.

**Fig. 3:**
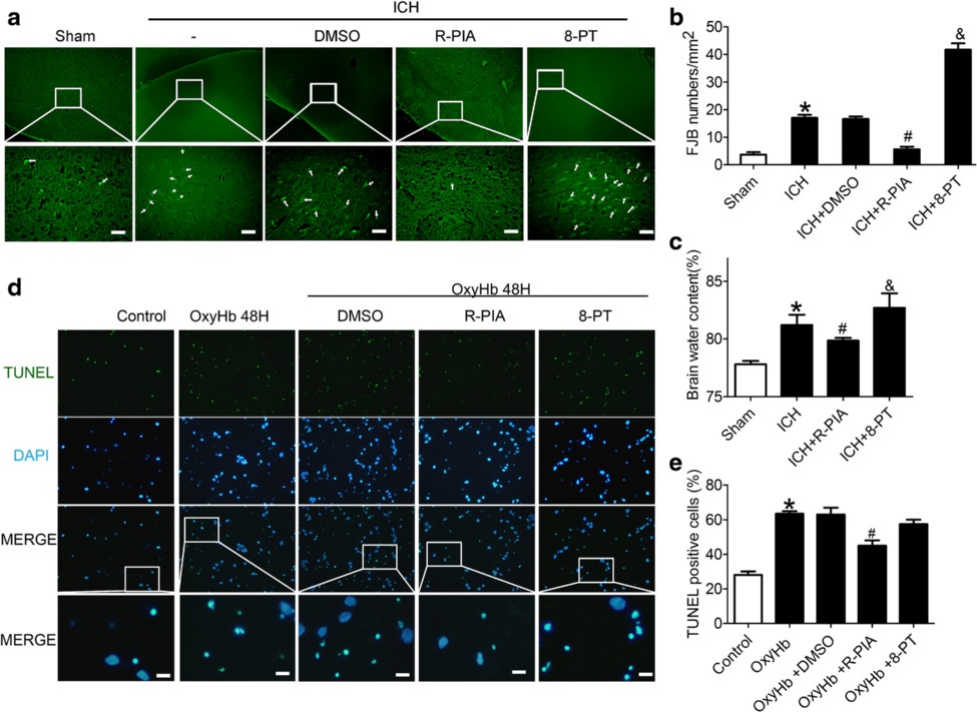
Changes in necrotic and apoptotic neurons, and brain water content after A1AR stimulation or inhibition. **a** FJB staining showing effects of A1AR on SBI at 48 h after ICH onsets. Representative images from sham, ICH, ICH + DMSO, ICH + R-PIA, and ICH + 8-PT groups. Each group was subjected to ICH except for the sham group. Scale bar = 50 μm. **b** Quantification of the FJB staining in each group. FJB-positive cells were counted per unit area. *p < 0.05 for the ICH group versus the sham group, ^#^p < 0.05 for the ICH + R-PIA group versus the ICH group, & p < 0.05 for the ICH + 8-PT group versus the ICH group. **c** Brain water content of sham, ICH, ICH + R-PIA, and ICH + 8-PT groups at 48 h after ICH onsets. *p < 0.05 for the ICH group versus the sham group, ^#^p < 0.05 for the ICH + R-PIA group versus the ICH group, & p < 0.05 for the ICH + 8-PT group versus the ICH group. **d** TUNEL staining to elucidate the role of A1AR in OxyHb-treated neurons in vitro. Representative images from control, OxyHb, OxyHb + DMSO, OxyHb + R-PIA, and OxyHb + 8-PT groups. Each group was subjected to OxyHb except for the control group. Scale bar = 20 μm. **e** The percentage of TUNEL-positive cells. *p < 0.05 for the OxyHb group versus the control group, ^#^p < 0.05 for the OxyHb + R-PIA group versus the OxyHb group

In addition, in ICH group, the number of FJB-positive cells clearly increased compared with the sham group. And the number of FJB-positive cells decreased significantly in the ICH + R-PIA group and increased significantly in the ICH + 8-PT group (Fig. 3a and b) (p < 0.05).

Brain water content was also examined at 48 h after ICH and showed a significantly higher brain water content in brain samples of ICH group compared with those in the sham group (Fig. 3c) (p < 0.05). And the mean value of brain water content was decreased after administration of the A1AR agonist R-PIA (p < 0.05). In contrast, after administration of the A1AR antagonist 8-PT, brain water content increased significantly. In addition, we performed TUNEL studies in vitro (Fig. 3d and e) and results were similar to those from in vivo experiments.

As described above, these results suggested that pharmacological activation targeting A1AR provided effective inhibition of brain injury that occur following ICH.

### A1AR increased phosphorylation levels of P38, MAPKAP2, and Hsp27

**Fig. 4:**
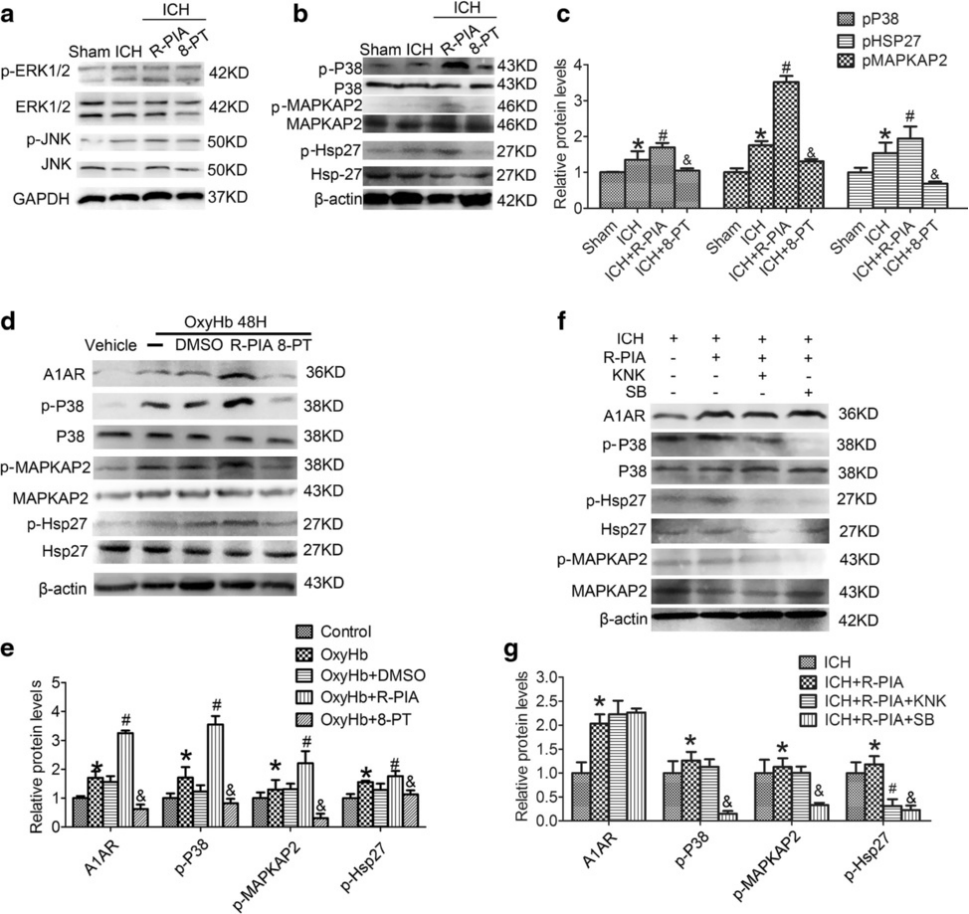
The mechanisms underlying A1AR effects in SBI. **a** Western blot analysis showing phosphorylation level of ERK1/2 and JNK in the sham, ICH, ICH + R-PIA, and ICH + 8-PT groups at 48 h after ICH onsets. **b** Western blot analysis showing expression of p-P38, p-Hsp27, and p-MAPKAP2 in the sham, ICH, ICH + R-PIA, and ICH + 8-PT groups. **c** Quantification of the results in panel **b**. The mean values of the protein levels in the sham group were normalized to 1.0. *p < 0.05 for the ICH group versus the sham group, ^#^p < 0.05 for the ICH + R-PIA group versus the ICH group, & p < 0.05 for the ICH + 8-PT group versus the ICH group. **d**, **e** Western blot analysis showing expression of p-P38, p-Hsp27, and p-MAPKAP2 in vitro. *p < 0.05 for the OxyHb group versus the control group, ^#^p < 0.05 for the OxyHb + R-PIA group versus the OxyHb group, & p < 0.05 for the OxyHb + 8-PT group versus the OxyHb group. **f** Following treatment with the P38 (SB203580) and Hsp27 (KNK437) antagonists, we detected changes in levels of p-P38, p-Hsp27, and p-MAPKAP2 in vivo at 48 h after ICH onsets. **g** Quantification of the results in panel **f**. The mean values of the protein levels in the ICH group were normalized to 1.0. *p < 0.05 for the ICH + R-PIA group versus the ICH group, ^#^p < 0.05 for the ICH + R-PIA group versus the ICH + R-PIA + KNK group, & p < 0.05 for the ICH + R-PIA group versus the ICH + R-PIA + SB group

To investigate the underlying mechanism by which A1AR prevent ICH-induced SBI and whether MAPK family participates in the process, the activation of ERK, p38 and JNK was tested by western blot analysis. Compared with sham group, the phosphorylation levels of ERK, p38 and JNK all were increased at 48 h after ICH onsets, suggesting ICH may induce the activation of ERK, p38 and JNK. In addition, R-PIA caused a statistically significant increase in phospho- p38, however, did not modify the phosphorylation of both ERK and JNK. These results showed that protection attained 48 h after ICH, by administration of A1AR agonists, involves increased activation of p38 MAPK (Fig. 4a-c).

Lee et al. have suggested that A1AR could exert a protective effect in transient renal ischemia through activation of the P38-MAPKAP2-Hsp27 pathway [31]. In this study, compared with the sham group, remarkable increases in the phosphorylation levels of MAPKAP2 and Hsp27 were observed in the ICH group (p < 0.05), which were further increased by R-PIA treatment and reversed by 8-PT(p < 0.05) (Fig. 4b and c). Similar results were also obtained in vitro (Fig. 4d and e).

### Blocking P38 or Hsp27 inhibited the neuroprotective effect of A1AR

**Fig. 5:**
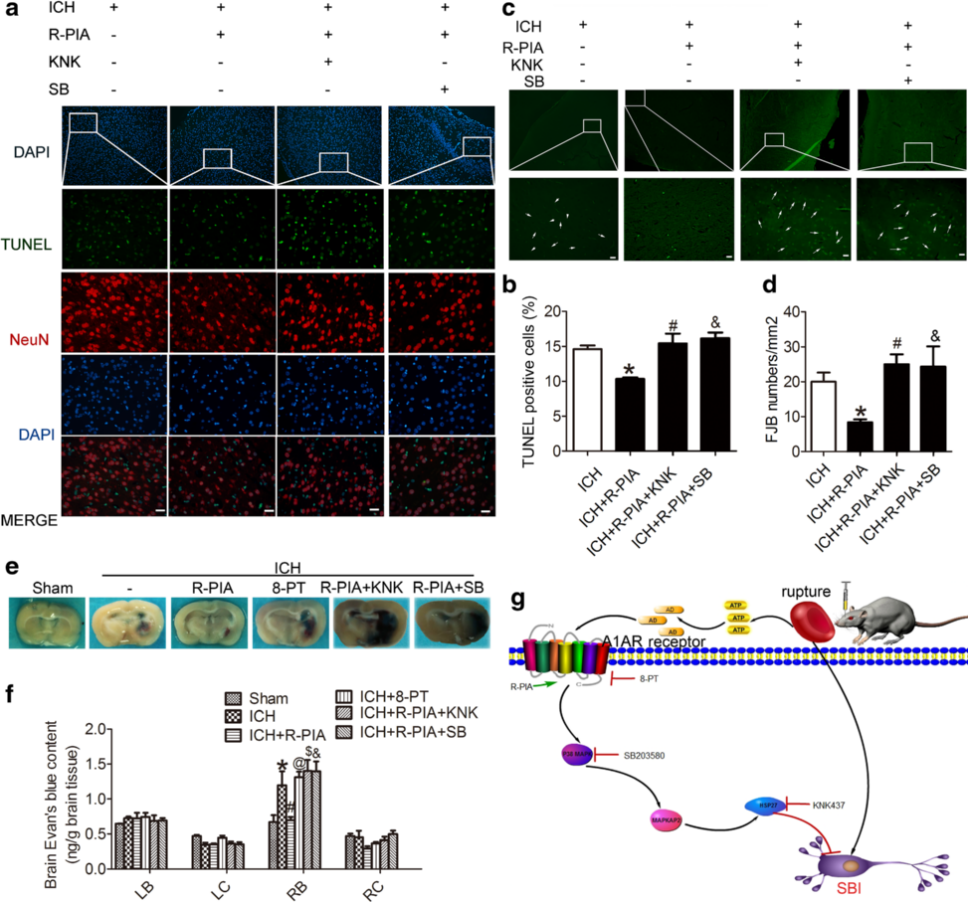
TUNEL, FJB, and EB results with antagonists for P38 and Hsp27, and proposed underlying mechanisms. **a** TUNEL staining showing apoptotic cells in ICH, R-PIA, Hsp27 antagonist (KNK437), and P38 antagonist (SB203580) groups **at** 48 h after ICH onsets. Each group was subjected to ICH. Double immunofluorescence analysis was performed with TUNEL (green) and a neuronal marker (NeuN, red), and nuclei were fluorescently labeled with DAPI (blue). Arrows indicate TUNEL-positive neurons. Scale bar = 50 μm. **b** The percentage of TUNEL-positive neurons in each group. *p < 0.05 for the ICH + R-PIA group versus the ICH group, ^#^p < 0.05 for the ICH + R-PIA group versus the ICH + R-PIA + KNK group, & p < 0.05 for the ICH + R-PIA group versus the ICH + R-PIA + SB group. **c** FJB staining showing FJB-positive cells in the ICH, R-PIA, Hsp27 antagonist (KNK437), and P38 antagonist (SB203580) groups at 48 h after ICH onsets. Each group was subjected to ICH. Scale bar = 50 μm. **d** Quantification of the FJB staining in each group. FJB-positive cells were counted per unit area. *p < 0.05 for the ICH + R-PIA group versus the ICH group, ^#^p < 0.05 for the ICH + R-PIA group versus the ICH + R-PIA + KNK group, & p ˂ 0.05 for the ICH + R-PIA group versus the ICH + R-PIA + SB group. **e**, **f** EB results for sham, ICH, ICH + R-PIA, ICH + 8-PT, ICH + R-PIA + KNK, and ICH + R-PIA + SB groups. *p < 0.05 for the ICH group versus the sham group, ^#^p < 0.05 for the ICH + R-PIA group versus the ICH group, ^@^ p < 0.05 for the ICH + 8-PT group versus the ICH group, ^$^p < 0.05 for the ICH + R-PIA + KNK group versus the ICH + R-PIA group, &p < 0.05 for the ICH + R-PIA + SB group versus the ICH + R-PIA group. G: Proposed mechanism for the role of A1AR in SBI

We further investigated the role of P38/MAPKAP2/Hsp27 in A1AR’s action using antagonists of p38 (SB203580) and Hsp27 (KNK437) (Fig. 4e and f). Following pretreatment with R-PIA before ICH, the phosphorylation levels of P38, MAPKAP2, and Hsp27 increased significantly compared with the ICH group (*p <* 0.05). After blocking Hsp27, the phosphorylation level of Hsp27 showed a significant decrease compared with the R-PIA group, while the phosphorylation levels of p38 and MAPKAP2 showed little change. After blocking p38, the phosphorylation levels of P38, MAPKAP2, and Hsp27 were all clearly decreased compared with the R-PIA group (p < 0.05) (Fig. 4f and g). In addition, in the presence of any of these antagonists, R-PIA -induced A1AR activation could not effectively suppress neuronal death and degeneration induced by ICH (Fig. 5a-d). Thus, we speculated that A1AR exhibited a neuroprotective function, which might act through the P38-MAPKAP2-Hsp27 pathway.

Finally, we used Evans blue (EB) to assess permeability of the blood–brain barrier (BBB). The content of EB exuded from blood vessels in different groups is shown in Fig. 5e and f. Compared with the sham group, EB content in the brain increased significantly in the ICH group (p < 0.05), which was significantly suppressed by R-PIA treatment and aggravated by 8-PT treatment (p < 0.05). And both SB203580 and KNK437 could abolish the protective effects of R-PIA on ICH-induced BBB dysfunction.

## Discussion and Conclusions

Although the A2AR has been the subject of most previous studies (see Introduction), our results show that the protein level of A1AR, but not A2A, A2B, or A3, was significantly increased after ICH. In addition, we have demonstrated that the A1AR agonist R-PIA could reduce ICH-induced SBI via modifying the activation of p38, but not ERK1/2 and JNK. Base on these results, we hypothesized here that adenosine is extensively generated from ATP released by ruptured red cell during ICH and quickly activates its receptor, A1AR at least partially, in neuron cells, and then elevated the activity of P38-MAPKAP2-Hsp27 pathway, which exerts neuroprotective effect and suppresses ICH-induced EBI (Fig. 5g).

As firstly put forward by Drury in 1929, the concept of purines as extracellular signaling molecules was approved. Extracellular purines (ATP, ADP, and adenosine) have been proved to be involved in diverse biological functions, including neurotransmission, smooth muscle contraction, exocrine and endocrine secretion, inflammation, the immune response, and platelet aggregation, via cell-surface receptors termed purine receptors. As early as minutes after ICH, extravasated blood components and damage-associated moleculars, such as ATP released from damaged and necrotic tissue, impose a pro-oxidative, strong cytotoxic, and proinflammatory insult toward adjacent viable brain cells [32]. And it has been reported that, both in the ATP-injected substantia nigra and cortex, ATP rapidly induced death of the neurons and astrocytes in the injection core area within 3 h [30, 33]. Given the above evidence, the cytotoxic effects of ATP tend to occur at the early stage of ICH-induced brain injury, which may be related to the narrow therapeutic window for the acute phase of ICH. Fortunately, the released ATP can be hydrolyzed extracellularly by a variety of cell surface-located enzymes referred to as ectonucleotidases. Extracellular ATP and ADP can be step by step hydrolyzed to adenosine by ectonucleotide pyrophosphatase/phosphodiesterases (E-NPPs), ectonucleoside triphosphate diphophohydrolases (E-NTPDases), and alkaline phosphatases (APs), while extracellular AMP is substrate of ecto-5′-nucleotidase (eN) and APs [34]. As the final product of hydrolysis cascade, adenosine and its receptors attracted more and more attention. In this study, we focused on the role of adenosine receptors in ICH-induced SBI and the underlying mechanisms.

There are 4 adenosine receptor subtypes: A1AR, A2aAR, A2bAR, and A3AR [35, 36]. Numerous authors indiscriminately select A2aAR, and Lin et al. have shown that an A2aAR agonist is effective in preventing vasospasm in subarachnoid hemorrhage conditions by inhibiting eNOS expression in brain vessels and inducing iNOS expression [37, 38]. However, in our time course experiment, A2aAR did not increase as much as A1AR after ICH. Thus, we selected A1AR for further study. In addition, as shown in Fig. 2a and b, the protein level of A1AR was increased to some extent under ICH, which is enhanced by its agonists R-PIA and inhibited by its antagonists 8-PT. Accordingly, R-PIA treatment alleviated ICH-induced SBI, which was aggravated by 8-PT treatment. The results are consistent with the previous report about the correlation between the efficacy and the expression of adenosine receptors [29] and suggest that ICH-induced A1AR activation maybe a self-help measure in neurons.

Many authors have suggested that A1AR activation has a beneficial effect [39–41]. Lee et al. indicated that endogenous A1AR activation produced cytoprotective effects in the renal proximal tubule, and Kim found that endogenous A1AR activation prevented murine liver ischemia reperfusion injury from worsening. However, others also have suggested that blocking A1AR exerted protection [42, 43]. In this study, we used R-PIA, a specific A1AR receptor agonist, to evaluate the role of A1AR in neuronal function under ICH conditions. At 48 h, the numbers of apoptotic or necrotic cells were clearly reduced in ICH groups pretreated with R-PIA (Fig. 2 and 3). A positive effect of 8-PT on apoptosis and necrosis was observed.

Lee demonstrated that endogenous A1AR activation produced cytoprotective effects by modulating the P38-MAPKAP2-Hsp27 pathway in renal cells [31]. However, no one has reported such effects in ICH conditions. In our experiment, inhibition of P38 suppressed the upregulation of phosphorylation of P38, MAPKAP2, and Hsp27, while the antagonist of Hsp27 (KNK437) clearly inhibited upregulation of Hsp27 phosphorylation. And both the two antagonists could almost completely abolish R-PIA- mediated neuroprotective effect (Fig. 5). Thus, these data suggest a crucial role for the P38-MAPKAP2-Hsp27 pathway in ICH conditions. In support of this concept, Carroll and Yellon have presented preliminary results from a cardiac myoblast cell line showing that pretreatment with P38 MAPK inhibitor SB203580 completely abolished delayed protection after ischemic- or adenosine-induced preconditioning [44].

Hsp27, a member of the heat shock protein family, is a molecular protein with diverse cytoprotective effects. Van Why et al. [45] have demonstrated that Hsp27, together with the actin cytoskeleton, restricts injury in vitro. The preservation of cytoskeletal actin contributes to architectural integrity of the cytoskeleton, at least in part. Phosphorylation of Hsp27 maintains the integrity of cells and enhances their ability to resist breaking down after ATP depletion [46]. In addition, Hsp27, acting as a molecular chaperone, prevents unfolded proteins from irreversible aggregation, reduces oxidative stress-mediated injury, and counteracts apoptosis by interacting with caspases [47, 48]. We propose that induction and increased phosphorylation of Hsp27 may provide protection against necrosis as well as apoptosis in neurons. In this study, we showed that activating A1AR with agonist R-PIA increased the phosphorylation level of Hsp27.

Finally, our experiments had limitations. The animals were intraperitoneally injected with A1AR agonist R-PIA and A1AR antagonist 8-PT. When these drugs crossed the BBB and finally reached the brain, their biological activity was limited. In addition, although there was no significant cardiovascular complications of R-PIA and 3-PT (see Methods), further pharmacological and toxicology experiment is still urgently needed to eliminate other potential side effects of them. In our experiment, we used healthy Sprague–Dawley (SD) rats to mimic the progression of ICH; however, in the clinic, we often see older patients. Finally, in vitro, we only treated neurons with oxyhemoglobin (OxyHb), and it remains to be determined if it is appropriate for activation of A1AR.

In conclusion, the present study demonstrated that, following ICH, the release of hematoma components, such as adenosine, may induce an increase in the protein level of A1AR, which maybe a self-help measure in neurons. A1AR agonist R-PIA could significantly increase the protein level of A1AR and alleviate ICH-induced SBI via P38-MAPKAP2-Hsp27 pathway, while A1AR antagonist 8-PT exerts opposite effects. We thus propose A1AR as a critical endogenous physiological regulator in neurons and suggest it may be potential therapeutic target in the treatment of ICH.

## Methods

### Study design and experimental groups

**Fig. 6:**
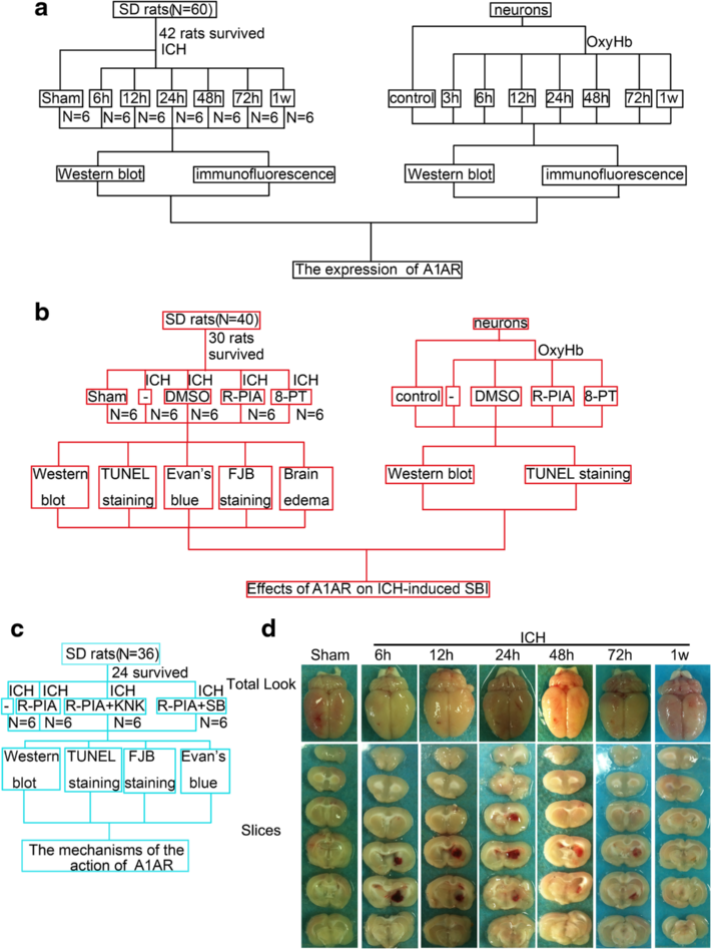
Experimental protocols. **a** Experiment 1 was designed to evaluate expression of A1AR. **b** Experiment 2 was designed to investigate the role of A1AR in ICH-induced SBI. **c** Experiment 3 was designed to elucidate mechanisms underlying the effects of A1AR. **d** Representative whole brains and brain slices from ICH model rats at each time point

Drug administration was shown in Fig. 6a-c. Briefly, R-PIA, prepared in normal saline containing 20 % DMSO at a concentration of 5 μg/ml, was injected intraperitoneally at a dose range of μg/kg body weight. 8-PT was prepared in normal saline containing 10 % DMSO at a concentration of 0.2 mg/ml and injected intraperitoneally at a dose of 3 mg/kg body weight. KNK was prepared in DMSO at a concentration of 2.5 μg/μl and injected intracerebroventricularly at a dose of 16.7 μg/kg body weight [49]. 5 μl SB203580 in normal saline containing 0.1 % DMSO at a concentration of 1 mM were given via intracerebroventricular injection [50]. All the drugs were administered 30 min before induction of ICH. To pursue the potential toxic side effects of all the four drugs, we have treated normal rats with them at the same dosage of that used in this study in preliminary experiments. No rat died in all the groups. Blood pressure and heart rate were measured via the cannulated right femoral artery. There were no significant differences in blood pressure and heart rate among vehicle group, R-PIA group, 8-PT group, KNK group, and SB203580 group (Data not shown).

### Antibodies

Anti-A1AR antibody (ab82477), anti-active caspase-3 (ab2302), Chk pAb to albumin (ab106582), Rb pAb to MAPKAP2 (ab131531), Rb pAb to phosphor MAPKAP2 (ab63378), Ms mAb to Hsp27 (ab2790), Rb pAb to phosphor Hsp27 (ab5594), Rb mAb to NeuN (ab177487), anti-ERK1 (phospho T202) + ERK2 (phospho T185) antibody (ab201015), anti-ERK1 + ERK2 antibody (ab17942), anti-JNK1 (phospho T183) antibody (ab47337), anti-JNK1 antibody (ab110724), anti-GFAP antibody (ab10062), and Ms mAb to NeuN (ab104224) were purchased from abcam. Anti-A2aAR antibody (sc-32261), anti-A2bAR antibody (sc-28996), anti-A3AR antibody (sc-13938), β-actin (sc-47778), and GAPDH (sc-365062) were purchased from Santa Cruz. Rb pAb to P38, phosphor P38 was purchased from Cell Signaling.

### Establishing the ICH model

Adult male SD rats (0–300 g) were anesthetized with 4 % chloral hydrate, which was injected intraperitoneally at a dose of 1 mL per 100 g. After the rat was fully anesthetized, it was fixed in a stereotactic apparatus frame (Shanghai Ruanlong Science and Technology Development Co., Ltd., Shanghai, China). Extra chloral hydrate was injected if needed based on responses to tail pinches. The rat was then placed supine on a heating pad, which maintained temperature at approximately 27–35 °C. The experimental ICH model was produced using stereotaxic insertion of a needle with a rounded tip and a side hole into the basal ganglia [51, 52]. The position of basal ganglia was 3.5 mm lateral to the midline, 0.2 mm posterior to bregma, and 5.5 mm ventral to the cortical surface. After the microinjector was in position, collagenase IV was injected over 5 min, and the needle was left in the brain for 5 min. Bone wax was used as needed to block the burr hole to prevent loss of CSF and blood from the midline vessels. Next, we sutured the scalp and returned the rat to its cage where it had free access to food and water. Representative brain slices from the ICH model are shown in Fig. 6d. Assessment of SBI was performed at 48 h after ICH onsets.

### Western blot analysis

After brain tissues were collected, the brain tissue from each rat was separately homogenized and lysed in ice-cold RIPA lysis buffer (P0013; Beyotime, Shanghai, China). After centrifuging at 12,500 × *g* for 15 min at 4 °C, the supernatants were collected. A standard BCA (Beyotime, P0012) method was used to determine protein concentration. Then, protein samples (100 mg/lane) were loaded onto a 10 % SDS polyacrylamide gel, separated, and electrophoretically transferred to a PVDF membrane (IPVH00010; Millipore, Billerica, MA, USA). The membrane was blocked in 5 % nonfat milk for 2 h at 37 °C. Next, the membrane was incubated with the primary antibody overnight at 4 °C and then with the horseradish peroxidase-linked secondary antibody for 1.5 h at 37 °C. The membrane was washed with PBST and visualized using enhanced chemiluminesence detection (3100 Mini; Clinx Science Instruments Co.). The relative quantities of proteins were analyzed using Image J software.

### Immunofluorescence microscopy

We performed double labeling for A1AR, A2aAR, A2bAR, and A3AR with NeuN, to detect expression of A1AR, A2aAR, A2bAR, and A3AR in neurons. The sections were incubated with primary antibody, including antibodies for A1AR, A2aAR, A2bAR, A3AR, and NeuN antibody-neuronal cell marker (all diluted 1:100), overnight at 4 °C. Then, secondary antibodies were added, followed by washing 3 times with PBS. Secondary antibodies were Alexa Fluor 488 donkey anti-rabbit IgG antibody and Alexa Fluor 555 donkey anti-mouse IgG antibody (Life Technologies, Carlsbad, CA, USA, 1:300 dilution). After a final wash, sections were coverslipped with an anti-fading mounting medium containing 4,6-diamino-2-phenyl indole (DAPI, SouthernBiotech, Birmingham, AL, USA). Normal rabbit IgG and normal mouse IgG were used as negative controls for immunofluorescence assays (data not shown). Sections were observed with a fluorescence microscope (Olympus, BX50/BX-FLA/DP70, Olympus Co., Japan). The relative fluorescence intensity was analyzed using Image J.

### TUNEL staining

Brain tissues embedded in paraffin were used for TUNEL staining. The sections were deparaffinized, dehydrated by heating at 75 °C in an oven for 60 min, and then rehydrated through xylenes and graded ethanol solutions to water. The sections were then incubated in Triton X-100 for 10 min. After 3 washes in PBS (5 min per wash), the sections were incubated with the TUNEL reaction mixture for 1 h at 37 °C. Sections were once again washed 3 times in PBS (5 min per wash). After the final wash, sections were coverslipped with an anti-fading mounting medium containing DAPI. The number of TUNEL-positive neurons in each millimeter length was counted carefully per sample. Cell counts from the brain were averaged to provide the mean value.

### FJB

Cell necrosis in brain tissue was detected by FJB. FJB procedures were identical to those for TUNEL. Sections were deparaffinized, dehydrated in an oven, rehydrated through xylenes and graded ethanol solutions to water, and permeabilized in 0.04 % Triton X-100. Sections were then incubated in FJB dye solution. The sections were visualized by a fluorescence microscope (Olympus BX50/BXFLA/DP70; Olympus). The FJB-positive cells were counted by an observer who was blind to the experimental groups. To evaluate the extent of cell necrosis, the necrotic index was defined as the average number of FJB-positive cells in each section counted in 6 microscopic fields (×400 magnification).

### Brain edema

Rats were randomly divided into 4 groups: sham, ICH, R-PIA (7.5 g dissolved in 1.5 mL DMSO and NaCl solution) [53], 8-PT(0.9 mg dissolved in 4 mL DMSO and NaCl solution) [54]. The index of brain edema was evaluated using the wet/dry method. Briefly, after brain tissue was removed, the samples were collected and weighed immediately (wet weight), dried at 100 °C for 72 h, and then weighed again (dry weight). The percentage of water content was calculated as [(wet weight-dry weight)/wet weight] × 100 %.

### EB detection

BBB injury was determined by EB (Sigma-Aldrich, St Louis, MO, USA) extravasation. Briefly, EB (2 % in Normal saline, 2 mL/kg) was injected into the rat through the femoral vein 48 h after ICH, and it was allowed to remain in circulation for 1 h. Animals were then reanesthetized and perfused using PBS to wash intravascular EB dye. A 3 mm of the brain tissue (coronal plane) were taken and it was divided into four parts: left cortical, right cortical, left basal, right basal. The samples were incubated in 5 % TCA and centrifuged at 15,000 rpm for 10 min. The supernatant was collected and mixed with ethanol. Absorbance at 620 nm was measured in a spectrophotometer.

### Primary neuron-enriched cultures

Whole brains of 1-day-old pups (from pregnant SD rats) were used to prepare primary neuron-enriched cultures. We tried as much as possible to minimize the number of pups used and their suffering. In brief, we first removed the meninges and blood vessels of the brain. The brain tissues were then digested with 0.% trypsin for 5 min, and the digestion was terminated by washing the tissue 3 times with PBS. The brain tissue suspension was centrifuged at 500 × *g* for 5 min, and the pellet was resuspended in DMEM/F12 medium containing 10 % heat-inactivated fetal bovine serum, 1 mM sodium pyruvate, 2 mM L-glutamine, 100 mM nonessential amino acids, 50 U/mL penicillin, and 50 mg/mL streptomycin (all from GIBCO, Carlsbad, CA, USA). Finally, cells were seeded in 150-cm^2^ culture flasks in fresh medium. Afterwards, half of the medium was changed every 2 d. In general, approximately 2 weeks after the initial seeding, a confluent monolayer of neurons was achieved. Neurons were separated from the brain tissue by shaking the flask for 4 h at 150 rpm, collected by centrifugation, and re-seeded in 12-well plates with fresh DMEM/F12 medium.

### Treatments of neurons

Enriched neurons were divided into 5 groups: control, OxyHb, OxyHb + vehicle (DMSO, Sigma-Aldrich), OxyHb + R-PIA (Sigma-Aldrich), and OxyHb + 8-PT (Sigma-Aldrich). Forty-eight hours after neuron re-seeding, cells were treated with OxyHb (at a final concentration of 10 μM), DMSO (volume equal to R-PIA and 8-PT), R-PIA (at a final concentration of 100 nM) [39], or 8-PT (at a final concentration of 100 nM) in fresh medium. After incubation for 48 h (37 °C, 5 % CO_2_), the cell medium was removed. Cells were then washed 3 times in PBS and fixed with 4 % paraformaldehyde.

### Statistical analysis

Values are presented as means ± SEM. SPSS 11.5 (SPSS Inc., Chicago, IL, U.S.) was used for statistical analysis. The Mann–Whitney *U* test was used to compare behavior and activity scores among groups. Statistical comparisons between groups were performed using one-way analysis of variance followed by either a Dunnett’s or a Tukey’s post hoc test, the former for comparisons to a single control group, the latter to compare across multiple groups. Mortality was compared with a w2 test. A probability of *P <* 0.05 was considered statistically significant.

## Abbreviations

ICH, intracerebral hemorrhage; SBI, secondary brain injury; ARs, adenosine receptors; A1AR, A1 adenosine receptor; R-PIA, N(6)-cyclohexyladenosine; 8-PT, 8-phenyl-1,3-dipropylxanthine; TUNEL, terminal deoxynucleotidyl transferase-mediated dUTP nick end labeling; FJB, Fluoro-Jade B; EB, Evans blue; BBB, blood–brain barrier; SD, Sprague–Dawley; OxyHb, oxyhemoglobin; ERK1/2, extracellular signal-regulated kinase; PI3-K/AKT, phosphatidylinositol 3-kinase
